# DIAGNOSIS AND TREATMENT OF POSTERIOR INTEROSSEOUS NERVE ENTRAPMENT: SYSTEMATIC REVIEW

**DOI:** 10.1590/1413-785220172501164801

**Published:** 2017

**Authors:** MARCO AURÉLIO DE MORAES, RUBENS GUILHERME GONÇALVES, JOÃO BAPTISTA GOMES DOS SANTOS, JOÃO CARLOS BELLOTI, FLÁVIO FALOPPA, VINÍCIUS YNOE DE MORAES

**Affiliations:** 1. Universidade de São Paulo, Escola Paulista de Medicina, Department of Orthopedics and Traumatology, São Paulo, SP, Brazil.

**Keywords:** Radial nerve, Nerve compression syndromes/diagnosis, Nerve compression syndromes/therapy, Evidence-based medicine.

## Abstract

Compressive syndromes of the radial nerve have different presentations. There is no consensus on diagnostic and therapeutic methods. The aim of this review is to summarize such methods. Eletronic searches related terms, held in databases (1980-2016): Pubmed (via Medline), Lilacs (via Scielo) and Google Scholar. Through pre-defined protocol, we identified relevant studies. We excluded case reports. Aspects of diagnosis and treatment were synthesized for analysis and tables. Quantitative analyzes were followed by their dispersion variables. Fourteen studies were included. All studies were considered as level IV evidence. Most studies consider aspects of clinical history and provocative maneuvers. There is no consensus on the use of electromyography, and methods are heterogeneous. Studies have shown that surgical treatment (muscle release and neurolysis) has variable success rate, ranging from 20 to 96.5%. Some studies applied self reported scores, though the heterogeneity of the population does not allow inferential analyzes on the subject. few complications reported. Most studies consider the diagnosis of compressive radial nerve syndromes essentially clinical. The most common treatment was combined muscle release and neurolysis, with heterogeneous results. There is a need for comparative studies***. Level of Evidence III, Systematic Review.***

## RESUMO

***As síndromes compressivas do nervo radial tem apresentação diversa. Não há consenso sobre métodos diagnósticos e terapêuticos. O objetivo desta revisão é sintetizar tais métodos. Este estudo se baseou no método das revisões sistemáticas da literatura. Busca eletrônica de estudos primários utilizando termos correlatos, realizada nas bases de dados (1980 a 2016): Pubmed (via medline), Lilacs (via Scielo) e Google Scholar. Através de protocolo pré-definido, identificou-se estudos relevantes. Não houve restrições de idioma. Excluiu-se relatos de caso. Aspectos do diagnóstico e tratamento foram sintetizados em tabelas. Análises quantitativas foram seguidas de suas variáveis de dispersão, considerando IC de 95%. Incluiu-se catorze estudos foram incluídos. Todos estudos foram considerados como nível IV de evidência. A maioria dos estudos consideram aspectos da história clínica e manobras provocativas como definidores de diagnóstico. Não há consenso sobre utilização da eletroneuromiografia, e os métodos são heterogêneos. Estudos demonstram que o tratamento cirúrgico (liberação muscular e neurólise) apresenta taxa variável de bons resultados, variando de 20-96,5%. Alguns estudos aplicaram escores autorreportados, entretanto a heterogeneidade das populações não permite análises inferenciais sobre o tema. Reportou-se poucas complicações. A maioria dos estudos consideram o diagnóstico da síndromes compressivas do nervo radial eminentemente clínicas. O tratamento cirurgico mais utilizado foi técnica mista de liberação muscular e neurólise, com resultados heterogêneos. Necessita-se de estudos comparativos. Nível de Evidência III, Revisão Sistemática.***

## INTRODUCTION

Compressive syndromes of the radial nerve present themselves in distinct ways; they can be purely sensory, motor or mixed.^1^ This group contains several syndromes such as radial tunnel syndrome, Wartenberg's syndrome, and posterior interosseous nerve syndrome. Because these syndromes affect different compression sites, they present distinct clinical conditions and potential diagnostic intersection with other conditions such as lateral epicondylitis of the elbow. The most common compressive syndromes are distal to the level of the elbow.^1^ ^,^ ^2^

Methods for diagnosing these injuries are based on clinical criteria but may also include imaging methods and electrophysiological studies.^2^ These diagnostic criteria vary widely because of both clinical maneuvers and quantitative criteria of imaging examinations.^2^ This fact makes the study of this group of diseases challenging because there is a lack of uniformity. Along similar lines, controversy exists with regard to treatment methods (conservative versus surgical) and there is no criteria for defining good or bad results.

Systematic literature reviews are secondary studies (utilizing research from primary studies) that bring together and synthesize data in order to summarize the stage of research on a given topic, standardize the current research, and propose strategies for future studies.^3^ This is an essential function to advance research on the topic, in addition to guiding those who are interested in the field.^4^

Using a systematic review, the objective of this study was to describe the clinical conditions (in adult patients), diagnosis, and treatment of compressive syndromes of the radial nerve, more specifically posterior interosseous nerve syndrome while excluding traumatic conditions and open injuries such as paralysis after humeral fracture.

## METHODS

The presentation of this systematic review follows the parameters of the PRISMA Statement,^5^ which regulates the methods of dissemination for systematic reviews and meta-analyses. This study was approved by the Institutional Review Board of the Universidade Federal de São Paulo under process number CEP 0108/2016.

The selected clinical studies were from adult patients with radial nerve entrapment syndromes, which aimed to present results of diagnosis and treatment. Studies conducted since 01/01/1980 were included, and there were no restrictions on language or with regard to follow-up time or temporality of the study (prospective/retrospective review). The following study designs were included: case series (retrospective/prospective study), case-control, cohort studies, and randomized clinical trials. We opted to exclude case reports.

The data were obtained from the Pubmed database (via Medline, searched in March 2015), the Lilacs database (via Scielo, searched in March 2016) and Google Scholar, using an active keyword search (MesH and non-MesH): Search terms were (Medline via Pubmed, Scielo, and Google Scholar): "radial tunnel syndrome" AND "radial neuropathy" AND "radial nerve palsy" AND "radial nerve neuritis" AND "posterior interosseous nerve neuropathy" AND "radial nerve neuropathy" AND "radial nerve palsy" and free search for the terms "radial nerve compression", "radial nerve syndrome", "Wartenberg syndrome", "radial nerve entrapment", "posterior interosseous compression". The operator OR was used for combinations of the terms in Pubmed. For Lilacs, an end-to-end study was used, and was limited to 01/01/1980. For relevant studies, we determined we would contact the study authors if there were any difficulties obtaining the data.

The studies were selected by assessing the title and structured abstract after selection in duplicate by two researchers (MM and RG). When there was disagreement between the researchers, a third researcher cast the tie-breaking vote (VM).

### Data extraction

The data were extracted using a protocol which was defined *a priori* and comprised the following information: description of the disease/condition, author, year of publication, journal, type of study, participants (inclusion and exclusion criteria), diagnostic criteria (criteria for clinical diagnosis, diagnostic criteria for supplementary examinations), intervention (clinical, surgical), results (symptoms cured/functional scores) and complications.

### Statistical analysis

After obtaining the data provided in this form, the authors gathered to summarize the data using qualitative tables (description of the condition, diagnostic methods, treatment methods, inclusion and exclusion criteria) and quantitative analysis via meta-analysis (risk/relative risk ratio and/or difference between the means). We chose to use REVMAN version 5.0 software for the quantitative analysis; the quantitative data were followed by their 95% confidence intervals. We chose to use a 5% alpha in the inferential analyses. The heterogeneity among the studies was assessed using l², and heterogeneity was considered high if l^2^ was greater than 75%.

## RESULTS

**Figure 1:**
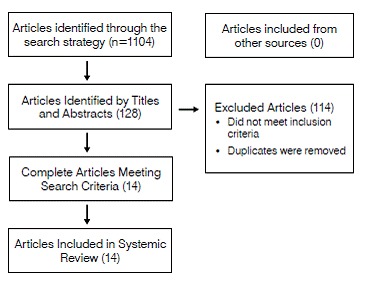
Flowchart of the search for studies.

We found 14 studies addressing the topic of this review.^6^ ^-^ ^19^ The flow chart (Figure 1) indicates the steps we took to obtain the included studies.

The demographic characteristics and the bibliometrics of the studies are presented in Tables 1 and 2.

**Table 1:** Characteristics of the included studies.

	Mean	Standard Deviation
Number of participants (n=14)	34.57	28.5
The number of limbs receiving intervention (n=14)	34.85	29.6
Time of follow-up (n=12)	86.08	72.8
Lost to follow-up (n=3)	7.3	2.5

**Table 2:** Characteristics of the studies: quantitative data.

Origin (n; %)	Type of journal (n; %)	Type of study (n; %)
Europe (7; 50%)	Hand surgery (3; 21.4%)	Case series, retrospective (7; 46.7%)
Asia (2; 14.2%)	Ortopedia (6; 42,8%)	Séries de caso, prospectiva (5; 33,4%)
Americas (5; 35.8%)	Electrophysiology (1; 7.1%)	Case series, prospective (5; 33.4%)
	Outras (4;28,5%)	Comparative study (case-control) (2; 3.4%)
	Other (4; 28.5%)	

In most studies, the clinical criteria were standardized, and some studies included eletroneuromyographic criteria. The majority included semiological aspects (principally pain) and semio-technical aspects (challenge via clinical maneuvers).

The results of the treatment, which for the most part are displayed qualitatively without focus on qualitative variables, showed high heterogeneity among the studies, with a discrepancy varying from 39 to 100% good results.

The proportions and confidence intervals, calculated for the purpose of this review, are shown in Table 3. Quantitative data were present in some studies,^10^ ^,^ ^11^ ^,^ ^14^ ^,^ ^15^ ^,^ ^17^ ^,^ ^18^and utilized the self-reported DASH/QuickDash and Roles & Maudsley tools. In general, the majority of studies described the open technique with muscle release associated with external neurolysis in the radial nerve. The exception that should be noted is the study by Lèclere et al.,^11^ who treated patients endoscopically.

**Table 3:** Characteristics of the studies: quantitative data.

Study	Proportion of good results	Confidence Interval
Hashizumi, 1996^9^	93%	n/a
Jebson, 1997^10^	48%	31%-65.5%
Leclère, 2012^11^	100%	n/a
Quignon, 2011^14^	60%	35.2%-84.8%
Rinker, 2004^15^	94%	n/a
Perez, 2014^17^	86%	75.7%-96.4%
Sotereanos, 1999^18^	39%	21.2%-75.4%

## DISCUSSION

In the absence of conclusive and well-delineated studies, the systematic reviews of non-randomized studies presented the following benefits: 1) they map the status of evidence, which is often deficient; 2) they determine failures in the process of determining scientific truths about the topic; 3) they quantify the available evidence, and 4) they propose and expose opportunities for future research, affirming the mobilization of human efforts and financial resources.

The results of this review demonstrated the poor quality of evidence on the subject. For the most part the studies were level IV evidence, susceptible to bias from memory and selection, and as a rule they tended to overestimate the effects of treatment and bias the sample of patients. Consequently, the results of this review are limited, but they do serve to illustrate the current state of research, ratifying and supporting new studies on the theme.

The results show that there is no uniformity in diagnosis and treatment, but the clinical criteria and treatment remained constant in some studies. In this respect, studies of accuracy (diagnostic properties of clinical maneuvers, electroneuromyography, and imaging examinations and comparison of treatment methods such as isolated muscle release, individual occurrence or association with neurolysis, and endoscopic methods) are a prolific area for science. This should, however, counterbalance the difficulty of grouping cohorts on this topic, because of its relatively low frequency and low rate of diagnosis.

Despite this lower frequency, the studies show a considerable rate of good results, but extrapolation of these results to practice can be subject to reference bias since the evaluator is not blinded, and there is no effective methodology for measuring outcomes, which should focus on patient-reported outcomes and measures of pain and recurrence. The literature exhibits a lack of studies reporting conservative treatment techniques, which leads us to raise the possibility that the cohorts in these studies are for the most part patients refractory to conservative methods of treatment. The small rate of complications, which could be the result of underreporting, should also be mentioned.

## FINAL CONSIDERATIONS

We found that the majority of studies consider diagnosis of compressive syndromes of the radial nerve to be eminently clinical. The most common treatment consisted of a mixed technique of muscle release and external neurolysis, with heterogeneous results. There is a need for comparative studies on the subject, ideally multi-center studies which examine the effectiveness of treatment methods and pay special attention to conservative methods of treatment.
